# Sharing the Power of White Privilege to Catalyze Positive Change in Academic Medicine

**DOI:** 10.1007/s40615-020-00947-9

**Published:** 2021-01-19

**Authors:** José E. Rodríguez, Dmitry Tumin, Kendall M. Campbell

**Affiliations:** 1 Office of the Associate Vice President for Health Equity, Diversity and Inclusion, Department of Family and Preventive Medicine, University of Utah Health, Salt Lake City, UT USA; 2 Division of Academic Affairs, Department of Pediatrics, Brody School of Medicine, East Carolina University, Greenville, NC USA; 3 Research Group for Underrepresented Minorities in Academic Medicine, Brody School of Medicine, Division of Academic Affairs, East Carolina University, 600 Moye Blvd AD-47, Greenville, NC 27834 USA

**Keywords:** Academic medicine, Equity, White privilege

## Abstract

White privilege can be often overlooked and poorly understood in academic medicine, by those who wield it, and by those who suffer from its deleterious effects. Dr. Peggy McIntosh, a leader in research on equity and diversity in education, described white privilege as a set of unearned benefits that white people have based on being born white in a culture that favors the white race. White people have privilege because it was given to them by other white people, and it was taken by claiming superiority over people of color, starting before the European colonizations of Africa, Asia, and the Americas, and continuing through the present day. Many white people come from impoverished communities, suffer from socioeconomic disadvantage, and struggle with unemployment. They may also suffer from inadequate housing and limited education. Because they are white, they still benefit from privilege and positive stereotypes associated with light skin color. As our nation reckons with the murders of unarmed Black people by police, recognizing that  many white people have been allies and agents of change forBlack and other minority people, discussing how the power of white privilege can be shared is needed. The authors discuss the power of white privilege and how that power can be shared to promote change in academic medicine.

## Introduction

Leaders in academic medicine are increasingly recognizing and reckoning with white privilege in their personal experience and in the operation of the institutions they inhabit [1, 2]. Dr. Peggy McIntosh, a leader in research on equity and diversity in education, and founder of the National “Seeking Educational Equity and Diversity” (SEED) Project on Inclusive Curriculum, described white privilege as a set of unearned benefits that white people have based on being born white in a culture that favors the white race [3]. White people have privilege because it was given to them by other white people. It was taken by claiming superiority over people of color starting before the European colonizations of Africa, Asia, and the Americas, and continuing through the present day [4, 5]. Although many white people come from impoverished communities, suffer from socioeconomic disadvantage, and struggle with unemployment, inadequate housing, and limited education, they still benefit from privilege and positive stereotypes associated with light skin color.

Among these positive stereotypes are seamless advancement without question because of white race (especially among males), attitudes of class superiority, and the “benefit of the doubt” based on social construct and phenotype. Internalized racism [6], the assumption that white is the norm and is desirable, is a result of centuries of white privilege setting up white race as a standard against which other groups are compared [6, 7]. Although white privilege is associated with power in today’s society—that is, the ability to command and direct valuable resources—the power conferred by white privilege is exclusive to one racial group. While recognizing the power associated with white privilege is important [8], a further step entails channeling this power into positive change through the purposeful actions of those who wield it.

Regardless of their individual background, leaders in academic medicine operate within an environment of institutional white privilege. Institutional white privilege assigns the assumptions and advantages of white privilege to institutions or organizations that are identified with white race. Like institutional racism, it unfairly favors some by disadvantaging others based on phenotype or social class [6]. For example, institutional white privilege asserts that predominantly white institutions (PWIs) are superior to historically black colleges and universities (HBCUs) and universities in Puerto Rico [9, 10]. Institutional white privilege is evident when hospitals and medical schools give white physicians greater respect, deference, resources, and tolerance of criticism than physicians from other groups [11]. Institutional white privilege also manifests as inequitable protection of academic freedom, where the ability to study and comment on all aspects of knowledge is stratified by race or ethnicity.

## Recognizing and Sharing the Power of White Privilege

Identifying white privilege is the first step towards sharing its power. The challenge in its identification is that people who possess it are often oblivious to its existence [3]. Raising the issues of racism and white privilege can elicit reactions of denial, hostility, anger, and violence [12]. To overcome or circumvent these responses, multi-pronged approaches to addressing systems of white privilege have been developed over the last several decades. These include bias and cultural competency training, implicit association testing, and workshops on restorative justice. These sorts of workshops and trainings have been tailored for many venues, including the clinical environment, classroom education, faculty and staff hiring, and medical school admissions [13–15]. Once the power of white privilege is identified, it can be defined,harnessed and ultimately shared. We propose that sharing the power of white privilege can be done in positive ways that improve the representation of underrepresented groups in medicine and increase the support provided to members of underrepresented groups. Specifically, institutional leaders in academic medicine can share the power of white privilege at the level of a medical school or academic medical center, leveraging their own white privilege to foster greater inclusion and equity at all levels of the organization.

To develop strategies for sharing the power of white privilege, we first map manifestations of individual privilege, taken from the work of Dr. Peggy McIntosh [3, 16], onto opportunities for individual and institutional action in academic medicine (Table 1). For example, the individual privilege of being able to work alongside people of the same race can be shared by leaders in position to make hiring decisions, who can aim to hire more people of color. The power associated with this privilege can also be shared through institutional actions, such as cluster hiring of underrepresented minority faculty. A further challenge entails sharing institutional white privilege, since power accruing to an entire privileged institution can be shared only through the actions of the institution as a whole.

**Table 1:** Spending assets to share individual white privilege

White privilege asset (modified from McIntosh 1989)	Individual steps to share the power of white privilege [16]	Institutional steps to share the power of white privilege
I can, if I wish at the workplace, arrange to be in the company of people of my race most of the time.	I try to diversify the groups I work with, promoting the hiring of more people of color.	Intentional underrepresented minority faculty cluster hiring.
I can speak in public to a powerful white male group without putting my race on trial.	I co-present on white privilege with persons of color.	Training to recognize impact of white privilege on underrepresented minority faculty, staff and students.
I can go home from most meetings of organizations I belong to feeling somewhat tied in, rather than isolated, out-of-place, outnumbered, unheard, held at a distance, or feared.	I listen and respond as an ally to people of color, acknowledging their contributions to mostly white organizations.	Highlighting and promoting accomplishments of underrepresented minority faculty; creating underrepresented minority affinity groups to reduce isolation; conducting training on allyship.
I am never asked to speak for all the people of my racial group.	I never assume that a person of color speaks for their whole racial group, and I explain that I speak only for myself.	Increasing underrepresented minority faculty representation to ensure multiple voices of color.
I can take a job with an affirmative action employer without having co-workers on the job suspect that I got the job because of my race.	I assume that everyone I work with has earned their position.	Viewing racial/ethnic diversity as an essential asset to the institution that improves teaching, scholarship, and health outcomes.

Specific institutional strategies to share the power of white privilege include PWIs initiating partnerships with minority-serving institutions [10, 17–19] to foster faculty collaboration as well as student growth and advancement. These partnerships can share privilege associated with PWIs’ historical advantage in financial resources and staffing. Recognizing that most medical school leaders are white men, who benefit from both white and male privilege, the leaders of PWI medical schools can collectively commit to lead and groom underrepresented minority faculty to take their place. In particular, they can intentionally coach and sponsor people with less power (underrepresented minorities) to take on key leadership roles, including senior leadership positions that have power and influence in the institution.

White privilege allows those who have it to ignore it, and it allows for experiences common to Black and other people of color to be overlooked. Personnel who participate in the application process at medical schools should explore the impact of social determinants of health, access to healthcare, and health outcomes of Black men [20], ensuring that allostatic load and ethno-historical trauma are addressed in medical education. A starting point would be learning about the damage white privilege can cause, including sources who speak of this from a white man’s perspective. [12, 21, 22] White privilege allows PWI leaders to insist on rigid “merit-based” admissions criteria, ensuring that admissions practices discriminate against applicants from disadvantaged backgrounds. It causes institutions to value accomplishments of applicants that are dependent on privilege. Faculty leaders can recognize this ingrained habit of blaming inequities (outcomes) on learners’ social, cultural, or educational backgrounds, and replace it with a practice of assigning academic value to life experience, including “distance traveled” for underrepresented minority students, as an essential component in a holistic admissions process. [23] This process should extend to undergraduate pre-medical advising, to overcome disparities in who is supported and encouraged to apply to medical school.

A common element of our suggestions is for academic medicine leaders to critically examine their institution’s policies and their own interactions with faculty and learners, asking themselves, “Are exclusionary practices operating here?” [24] Because white privilege allows current leadership of PWI medical schools to guide the direction of medical education in the future, leaders of these institutions can foster the elimination of entrenched biases, stereotypes, and discrimination. They can practice intentionality and critically examine and eliminate racist structures, policies, practices, embedded norms, and values that sustain inequities. In the near term, intentional conscious sharing of white privilege can improve institutions in many ways, from education, to patient care, to health outcomes. In the long term, the strategies we outline can become part of a blueprint for change in academic medical centers throughout the USA.

## Conclusion

Sharing the power of white privilege must entail a discussion that connects academic institutions and health centers nationwide. The power associated with white privilege can only be shared by those who have access to it. Underrepresented minority faculty voices, for all of their scholarship and accomplishments, cannot claim this power [1, 12]. Because academic medical institutions are mostly led by white men, who oftentimes will not talk about white privilege, these institutions also benefit from the power of white privilege. In this article, we proposed examples of how individuals and institutions can share the power of white privilege and move academic medicine towards greater equity. Arriving at a place of dialog, or a space where racism and privilege can be discussed without anger, resentment, or disbelief, will be an ongoing challenge to this effort. Medical school leaders can champion these discussions and ensure that resulting actions are measurable and sustainable.

While the power of white privilege is both an institutional and individual asset, it is not the only asset that individuals can use for change. As stated above, the power of white privilege gets its power from the denial of its existence. Using white privilege is difficult, and only when those who have it can talk about it freely can we make true change. For this reason, we admit that this path may not be the first choice for leaders to pursue, as opposed to “race neutral” policies that engender less controversy. Nevertheless, decisive action among leaders in academic medicine can drive culture change at academic medical centers. Many institutions already endorse the need to work across racial and ethnic differences to promote a more inclusive environment in academic medicine. In addition, some organizations are supporting projects to increase representation of minority faculty and including addressing historical injustices to promote inclusion [25]. Because minority representation in medicine has lagged behind minority representation in the general population, with few meaningful gains over the last several decades, new strategies are needed to address this disparity. Recognizing and using the power of white privilege could be the catalyst needed to accelerate this movement towards parity and equity.
