# Correction to: Sharing the Power of White Privilege to Catalyze Positive Change in Academic Medicine

**DOI:** 10.1007/s40615-021-01002-x

**Published:** 2021-03-15

**Authors:** José E. Rodríguez, Dmitry Tumin, Kendall M. Campbell

**Affiliations:** 1 Office of the Associate Vice President for Health Equity, Diversity and Inclusion, Department of Family and Preventive Medicine, University of Utah Health, Salt Lake City, UT USA; 2 Division of Academic Affairs, Department of Pediatrics, Brody School of Medicine, East Carolina University, Greenville, NC USA; 3 Research Group for Underrepresented Minorities in Academic Medicine, Brody School of Medicine, Division of Academic Affairs, East Carolina University, 600 Moye Blvd AD-47, Greenville, NC 27834 USA

**Correction to: J. Racial and Ethnic Health Disparities.**

10.1007/s40615-020-00947-9

The article “Sharing the Power of White Privilege to Catalyze Positive Change in Academic Medicine”, written by José E. Rodríguez, Dmitry Tumin, and Kendall M. Campbell, was originally published electronically on the publisher’s internet portal on 19 January 2021 without open access. With the author(s)’ decision to opt for Open Choice the copyright of the article changed on 26 January 2021 to © The Author(s) 2021 and the article is forthwith distributed under a Creative Commons Attribution 4.0 International License, which permits use, sharing, adaptation, distribution and reproduction in any medium or format, as long as you give appropriate credit to the original author(s) and the source, provide a link to the Creative Commons license, and indicate if changes were made. The images or other third party material in this article are included in the article’s Creative Commons license, unless indicated otherwise in a credit line to the material. If material is not included in the article’s Creative Commons license and your intended use is not permitted by statutory regulation or exceeds the permitted use, you will need to obtain permission directly from the copyright holder. To view a copy of this license, visit http://creativecommons.org/licenses/by/4.0/.

